# Association between autonomic control indexes and mortality in subjects admitted to intensive care unit

**DOI:** 10.1038/s41598-018-21888-8

**Published:** 2018-02-22

**Authors:** Alberto Porta, Riccardo Colombo, Andrea Marchi, Vlasta Bari, Beatrice De Maria, Giovanni Ranuzzi, Stefano Guzzetti, Tommaso Fossali, Ferdinando Raimondi

**Affiliations:** 1 Department of Biomedical Sciences for Health, University of Milan, Milan, 20133 Italy; 2 Department of Cardiothoracic, Vascular Anesthesia and Intensive Care, IRCCS Policlinico San Donato, San Donato Milanese, Milan, 20097 Italy; 3 Department of Emergency, L. Sacco Hospital, Milan, 20157 Italy; 4 Department of Electronics Information and Bioengineering, Politecnico di Milano, Milan, 20133 Italy; 5 IRCCS Istituti Clinici Scientifici Maugeri, Istituto di Milano, Milan, 20138 Italy; 6 Department of Anesthesia and Intensive Care, IRCCS Humanitas Clinical and Research Center, Rozzano, 20089 Italy

## Abstract

This study checks whether autonomic markers derived from spontaneous fluctuations of heart period (HP) and systolic arterial pressure (SAP) and from their interactions with spontaneous or mechanical respiration (R) are associated with mortality in patients admitted to intensive care unit (ICU). Three-hundred consecutive HP, SAP and R values were recorded during the first day in ICU in 123 patients. Population was divided into survivors (SURVs, n = 83) and non-survivors (NonSURVs, n = 40) according to the outcome. SURVs and NonSURVs were aged- and gender-matched. All subjects underwent modified head-up tilt (MHUT) by tilting the bed back rest segment to 60°. Autonomic control indexes were computed using time-domain, spectral, cross-spectral, complexity, symbolic and causality techniques via univariate, bivariate and conditional approaches. SAP indexes derived from time-domain, model-free complexity and symbolic approaches were associated with the endpoint, while none of HP variability markers was. The association was more powerful during MHUT. Linear cross-spectral and causality indexes were useless to separate SURVs from NonSURVs, while nonlinear bivariate symbolic markers were successful. When indexes were combined with clinical scores, only SAP variance provided complementary information. Cardiovascular control variability indexes, especially when derived after an autonomic challenge such as MHUT, can improve mortality risk stratification in ICU.

## Introduction

Various types of signal processing tools have been devised and applied to spontaneous fluctuations of cardiovascular variables recorded from short periods of time (i.e. about 5 minutes) in the attempt to characterize short-term cardiovascular control mechanisms^1,2^. Applications of these methods have been particularly successful in describing specific aspects of the cardiovascular control that are hardly quantifiable, such as vagal modulation^3^, or can be quantified with difficulty, such as cardiac baroreflex sensitivity^4^. The clinical utility of computing autonomic nervous system indexes in predicting the outcome of both cardiovascular and non-cardiovascular pathologies^5–14^ and in stratifying the risk for adverse perioperative and postoperative cardiac events^15–21^ has been proven by several studies. The clinical value of autonomic and cardiovascular control markers as risk predictors was confirmed in the emergency department^22,23^ and critical care unit (ICU)^24–28^. However, it is unclear whether the application of an autonomic challenge in critical clinical settings might improve further their predictive and discriminative power.

The aim of this study is to test the association of autonomic control parameters derived from spontaneous fluctuations of cardiovascular variables recorded within the first day after admission in ICU with mortality in critically ill patients and their incremental value to traditional clinical scores in connection with an autonomic challenge. A large sample of techniques, commonly applied to the analysis of short beat-to-beat variability series of heart period (HP) and systolic arterial pressure (SAP) and their link with respiration (R), were applied including linear and nonlinear univariate tools performing time-domain, frequency-domain, complexity and symbolic analyses^29–32^ and linear and nonlinear bivariate methods assessing HP-SAP and HP-R interactions regardless of their directionality^33–35^ or accounting for directionality^36,37^. An orthostatic challenge, namely the modified head-up tilt (MHUT)^38^, was employed to evoke a sympathetic activation in response to a central hypovolemia, thus challenging the autonomic control of critically ill patients.

## Materials and Methods

### Experimental protocol

We considered 140 critically ill patients (min-max range, age: 18–86 years, body mass index: 12–42 kg·m^−2^, 93 men) with an expected stay in ICU longer than 48 hours. The study was performed according to the Declaration of Helsinki for medical research involving humans. The protocol and all methods applied in the study were approved by the ethical review board of both “L. Sacco” Hospital, Milan, Italy and Humanitas Clinical and Research Hospital, Rozzano, Italy. The protocol was registered at ClinicalTrials.gov (NCT01930669 on August 14, 2013). All conscious patients gave their written informed consent. Close relatives or legal representatives of unconscious patients provided written informed consent. Exclusion criteria were age below 18 years, elective surgery patients admitted to ICU as a result of the standard postoperative protocol, non-sinus rhythm, presence of a significant amount of ectopic beats (>5%), spinal or head injury, suspected or documented intracranial hypertension, contraindications of any kind to 60° head-up tilt. Seventeen patients were a posteriori excluded because of sustained arrhythmias or poor signal quality, thus the analysis was performed over 123 patients. Surface electrocardiogram (ECG) and invasive arterial pressure were acquired from the patient’s monitor in the ICU (IntelliVue MX800 Patient Monitor, Philips, Best, The Netherlands). Signals were sampled at 250 Hz by using an analog-to-digital acquisition board (NI 9215, National Instruments, Austin, TX, USA). Signals were recorded at rest in supine position (REST) and during MHUT^38^ performed on the standard ICU three-segment bed (Total Care, Hill-Rom Company, Batesville IN, USA). During the MHUT session the patients’ bed was first tilted to 15° as a rigid body, then the inclination of back rest was increased to reach 60°, while the inclination of the thigh rest was adjusted to the horizontal position (i.e. 0°). The inclination of the shank rest was left to 15°. Both sessions lasted 10 minutes with MHUT always following REST. Experimental sessions were carried out during the patient’s first day in ICU. All patients were able to complete the overall protocol without experiencing any sign of pre-syncope. Based on the patient’s outcome starting from their admission in the ICU, the 123 patients were classified in survivors (SURVs, n = 83) and nonsurvivors (NonSURVs, n = 40).

### Extraction of the beat-to-beat variability and preprocessing techniques

After detecting the QRS complex on the ECG and locating with minimum jitters its peak using parabolic interpolation^39^, the temporal distance between two consecutive QRS complex apexes was computed and utilized as an approximation of the *n*th HP (HP_*n*_). The maximum of arterial pressure within HP_*n*_ was taken as the *n*th SAP (SAP_*n*_). The amplitude of the first R-wave delimiting the HP_*n*_ and measured from the isoelectric line provided the *n*th measure of R (R_*n*_), as derived from the respiratory-related amplitude modulations of the ECG^40^. Fiducial points were carefully checked to avoid erroneous detections or missed beats. If isolated ectopic beats affected HP and SAP values, these measures were linearly interpolated using the closest values unaffected by ectopic beats. Sequences of about 300 consecutive HP, SAP and R values were randomly selected inside REST and MHUT sessions. From HP and SAP series we computed in time domain the HP and SAP means, μ_HP_ and μ_SAP_. μ_HP_ and μ_SAP_ were expressed in ms and mmHg respectively. Then, HP, SAP and R series were linearly detrended before computing any additional cardiovascular variability index including HP and SAP variances termed σ^2^_HP_ and σ^2^_SAP_. σ^2^_HP_ and σ^2^_SAP_ and expressed in ms^2^ and mmHg^2^ respectively. No additional preprocessing techniques were applied except for model-based complexity, model-based and model-free causality analyses requiring the division of the zero-mean HP, SAP and R series by their standard deviation to avoid any dependence of complexity and causality indexes on the amplitude of the spontaneous fluctuations.

### Model-based spectral analysis

Power spectral analysis was carried out via a parametric method grounded on the identification of the coefficients of the autoregressive (AR) model and on a procedure decomposing the AR process into its basic components (see Supplement). The model order was optimized according to the Akaike figure of merit^41^ in the range from 8 to 14. The power was computed in the low frequency (LF) band (i.e. 0.04–0.15 Hz) and in the high frequency (HF) band (i.e. 0.15–0.4 Hz)^1^. We computed the HF power of HP series (HF_HP_) and the LF power of SAP series (LF_SAP_) both expressed in absolute units (i.e. ms^2^ and mmHg^2^ respectively) as indexes of vagal and sympathetic modulation directed to the heart and vessels respectively^3,29^. We computed the HF power of the R series expressed in percent units (HF%_R_) defined as the power of the R series in the HF band divided by the overall variance and the result multiplied by 100. Over the R series we estimated the respiratory rate (i.e. f_R_) as the frequency of the dominant component in the HF band. f_R_ was expressed in Hz.

### Model-based and model-free complexity analyses

Complexity analysis was carried out through the computation of conditional entropy (CE) via a linear model-based and a nonlinear model-free approach (see Supplement). The model-based CE (MBCE) was again grounded on fitting HP and SAP series with an AR model^32^. The identification procedure and model order selection were carried out as for spectral analysis. As a quantity linked to the CE but computed via a nonlinear model-free method, we utilized the sample entropy (SampEn)^30^. At difference with MBCE, SampEn was able to account for the eventual presence of nonlinear dynamical features present in the series. SampEn was computed according to standard settings (i.e. embedding dimension d = 3, tolerance r = 0.2 × standard deviation of the series, lag between consecutive samples forming the pattern τ = 1, and Euclidean norm to calculate distances among patterns).

### Univariate symbolic analysis

Univariate symbolic analysis was carried out according to a method^31^ using a uniform quantization approach over 6 bins, forming symbolic patterns by concatenating 3 consecutive symbols and grouping patterns into 4 families based on the number and sign of variations between adjacent symbols (see Supplement). The 4 pattern families are: i) no variation (0 V); ii) one variation (1 V); iii) two like variations (2LV); iv) two unlike variations (2UV). The percentage of 0 V, 1 V, 2LV and 2UV patterns (i.e. 0 V%, 1 V%, 2LV% and 2UV%) was obtained by dividing the number of patterns belonging to a class by the total number of patterns and, then, by multiplying the result by 100. Indexes were computed over HP and SAP series and labeled as 0 V%_HP_, 1 V%_HP_, 2LV%_HP_, 2UV%_HP_ and 0 V%_SAP_, 1 V%_SAP_, 2LV%_SAP_, 2UV%_SAP_ respectively.

### Model-based cross-spectral analysis

Cross-spectral analyses between HP and SAP and between HP and R were performed to estimate linear HP-SAP and HP-R dynamical relations. Cross-spectral analysis was carried out via a parametric method grounded on bivariate AR model (see Supplement). The model order was fixed^33^ to 10. HP-SAP cross-spectral analysis allowed us to compute the transfer function from SAP to HP indicated as H_HP-SAP_(f) and the squared coherence function between HP and SAP indicated as K^2^_HP-SAP_(f). The averaged modulus of H_HP-SAP_(f) in the LF band, indicated as |H_HP-SAP_(LF)|, was taken as an estimate of baroreflex sensitivity in the LF band^34,42^. |H_HP-SAP_(LF)| was expressed in ms·mmHg^−1^. The averaged K^2^_HP-SAP_(f) in the LF band, labeled as K^2^_HP-SAP_(LF) was taken as an estimate of the strength of the linear association between HP and SAP^43^. We computed also the averaged phase of H_HP-SAP_(f) in the LF band, denoted as PhH_HP-SAP_(LF). PhH_HP-SAP_(LF) was expressed in radians (rad). Negative phase values suggested that HP changes lagged behind SAP variations. The computation of |H_HP-SAP_(LF)| was performed without checking the prerequisites^34^ of significant K^2^_HP-SAP_(LF) and negative PhH_HP-SAP_(LF). An analogous analysis was carried out to characterize the HP-R dynamical relation in the HF band. We computed |H_HP-R_(HF)|, K^2^_HP-R_(HF) and PhH_HP-R_(HF) taken respectively as markers of the gain, strength and phase of the cardiorespiratory relation^44^. Negative PhH_HP-R_(HF) values suggested that HP changes lagged behind R fluctuations.

### Bivariate symbolic analysis

To account for the eventual presence of nonlinear interactions between two series, the bivariate joint symbolic analysis^35^ was applied over HP and SAP and over HP and R series. The bivariate joint symbolic analysis method exploited the univariate symbolic approach described in the Section “Univariate symbolic analysis” to build symbolic patterns over the two series (see Supplement). Then, joint symbolic schemes were formed by associating a pattern of one series with the pattern of the other starting τ cardiac beats ahead. Given the fastness of the baroreflex and cardiopulmonary interactions τ was set to 0 when both the dynamical interactions between HP and SAP and between HP and R were studied^45^. After defining as coordinated scheme as the one associating two patterns belonging to the same pattern family (i.e. 0V-0V, 1V-1V, 2LV-2LV and 2UV-2UV), their number was counted and their percentage over the class of coordinated patterns was tracked and indicated as 0V-0V%, 1V-1V%, 2LV-2LV% and 2UV-2UV%.

### Model-based Granger causality (MBGC) analysis

We exploited a traditional linear MBGC approach in the time domain (see Supplement) to assess the strength of the interactions from an input signal to an output one, while disambiguating the effect of a third one^46^. An AR model with two exogenous inputs was set in Ω = {HP,SAP,R} taking the HP as a target signal and SAP and R as exogenous ones. The variance of the HP prediction error in Ω was compared to the variance of the prediction error in Ω after excluding SAP through the log-causality ratio (logCR) (see Supplement). This index was labeled as logCR_SAP_ _→_ _HP_. The larger the logCR_SAP_ _→_ _HP_, the greater the involvement of cardiac baroreflex^37^. An analogous index compared the variance of the HP prediction error in Ω with the variance of the prediction error in Ω after excluding R and was denoted as logCR_R_ _→_ _HP_. The larger the logCR_R_ _→_ _HP_, the greater the strength of the cardiopulmonary coupling^37^. The delays from SAP to HP and from R to HP were set to 0 beats to account for the fast vagal arm of baroreflex and cardiopulmonary pathway^45^. The model order was optimized in Ω in the range from 4 to 14 according to the Akaike figure of merit for multivariate processes^41^ and, then, maintained in the restricted Ω obtained after excluding the presumed cause, while the coefficients of the model were identified again.

### Model-free Granger causality (MFGC) analysis

MFGC approaches^47^ extended the traditional MBGC technique^46^ by avoiding the *a priori* decision about the structure of the model and accounting for the eventual presence of nonlinear relations among signals in Ω. In Ω = {HP,SAP,R} we exploited the k-nearest neighbors approach^48^ with a zero order local approximation^36^ in a nonuniform embedding space^49,50^ built incrementally according to a procedure maximizing the correlation between the original series and the predicted one^36^. The variance of the prediction error in the restricted Ω was computed after excluding from the optimal multivariate embedding space built in full Ω the components relevant to the presumed cause. The same indexes as those computed via MBGC method describing in the Sect. “Model-based Granger causality (MBGC) analysis” were computed (i.e. logCR_SAP_ _→_ _HP_ and logCR_R_ _→_ _HP_). The number of nearest neighbors *k* was fixed to 60. The image of the reference vector was excluded from the set of images of the k nearest neighbors utilized to predict the current values to limit overfitting. At difference from the MBGC approach the delays from SAP to HP and from R to HP were optimized according to the procedure exploited to construct the multivariate embedding space^36^. The maximum multivariate embedding dimension was fixed to 15 and the maximum number of lagged variables considered over the same signal was set to 10.

### Statistical analysis

Population characteristics reported in Table 1 were tested according to χ2 test in the case of categorical variables and Mann-Whitney rank sum test in the case of continuous variables. Two-way repeated measures analysis of variance (one factor repetition, Holm-Sidak test for multiple comparisons) was used to check whether indexes exhibited between-group differences within the same experimental condition (i.e. REST or MHUT) and between-condition differences within the same group (i.e. SURV or NonSURV). Univariate Cox regression analysis was carried out by considering mortality as an outcome and the time elapsed from the admission to ICU to death for NonSURVs or to hospital discharge for SURVs, expressed in days, as independent time variable. Univariate Cox regression analysis was carried out over all variables, including the clinical ones that were found able to distinguish SURVs from NonSURVs. Those parameters found to be associated with mortality entered in a multivariate Cox regression model to assess their complementary value. Statistical analysis was carried out using a commercial statistical program (SPSS 22, IBM, Chicago, IL, USA). A *p* < 0.05 was always considered as significant.

**Table 1:** Population characteristics.

Variable	SURV (n = 83)	NonSURV (n = 40)
Age, years	64.1 (50.5–70.4)	62.8 (49.7–74.0)
Gender, male/female	52/31 (62.7/37.3)	30/10 (75.0/25.0)
BMI, kg·m^−2^	25.5 (23.5–27.8)	24.1 (21.9–27.3)
Ventilatory support, yes/no	68/15 (81.9/18.1)	37/3 (92.5/7.5)
Administration of catecholamines, yes/no	40/43 (48.2/51.8)	23/17 (57.5/42.5)
Sedation, yes/no	55/28 (66.3/33.7)	28/12 (70.0/30.0)
Septic shock, yes/no	15/68 (18.1/81.9)*	14/26 (35.0/65.0)
SOFA score	7.0 (6.0–10.0)*	11.0 (7.0–13.0)
SAPS II	39.0 (32.0–49.0)*	48.0 (36.0–55.5)
RASS score	–4.0 (−5.0 - –1.0)	–4.0 (−5.0 - –3.0)
LOS ICU, days	8.0 (5.0–13.0)	9.0 (6.0–18.0)
Intra-ICU mortality, yes/no	0/83 (0/100)*	28/12 (70.0/30.0)

ICU = intensive care unit; SURV = patient who survived; NonSURV = patient who did not survived; BMI = body mass index; SOFA = sepsi-related organ failure assessment; SAPS II = simplified acute physiology score; RASS = Richmond agitation-sedation scale; LOS = length of stay. Categorical variables are presented as count (percentage). Continuous variables are presented as median (first – third quartiles). The symbol *indicates a significant difference with *p* < 0.05 using χ^2^ for categorical variables and Mann-Whitney rank sum test for continuous variables.

## Results

Causes of patients’ admission to ICU were: respiratory failure (n = 69), septic shock (n = 25), postanoxic coma (n = 13), mix origin shock (n = 13), cardiogenic shock (n = 7), acute kidney failure (n = 4), infective origin coma (n = 3), cerebral injury coma (n = 2), acute lung injury/acute respiratory distress syndrome (n = 2), acute liver failure (n = 2). HP and SAP variability were reliably extracted from 123 patients divided in 83 SURVs and 40 NonSURVs (Tab.1). The two groups were age-, gender-, body mass index-, ventilatory support-, catecholamine administration-, and sedation-matched and had similar length of stay in ICU and Richmond agitation-sedation scale (Tab.1). As expected, NonSURVs had higher intra-ICU mortality (Tab.1). Moreover, NonSURVs were more frequently in septic shock and had significantly higher sepsi-related organ failure assessment (SOFA) score and simplified acute physiology score (SAPS II) (Tab.1).

**Figure 1:**
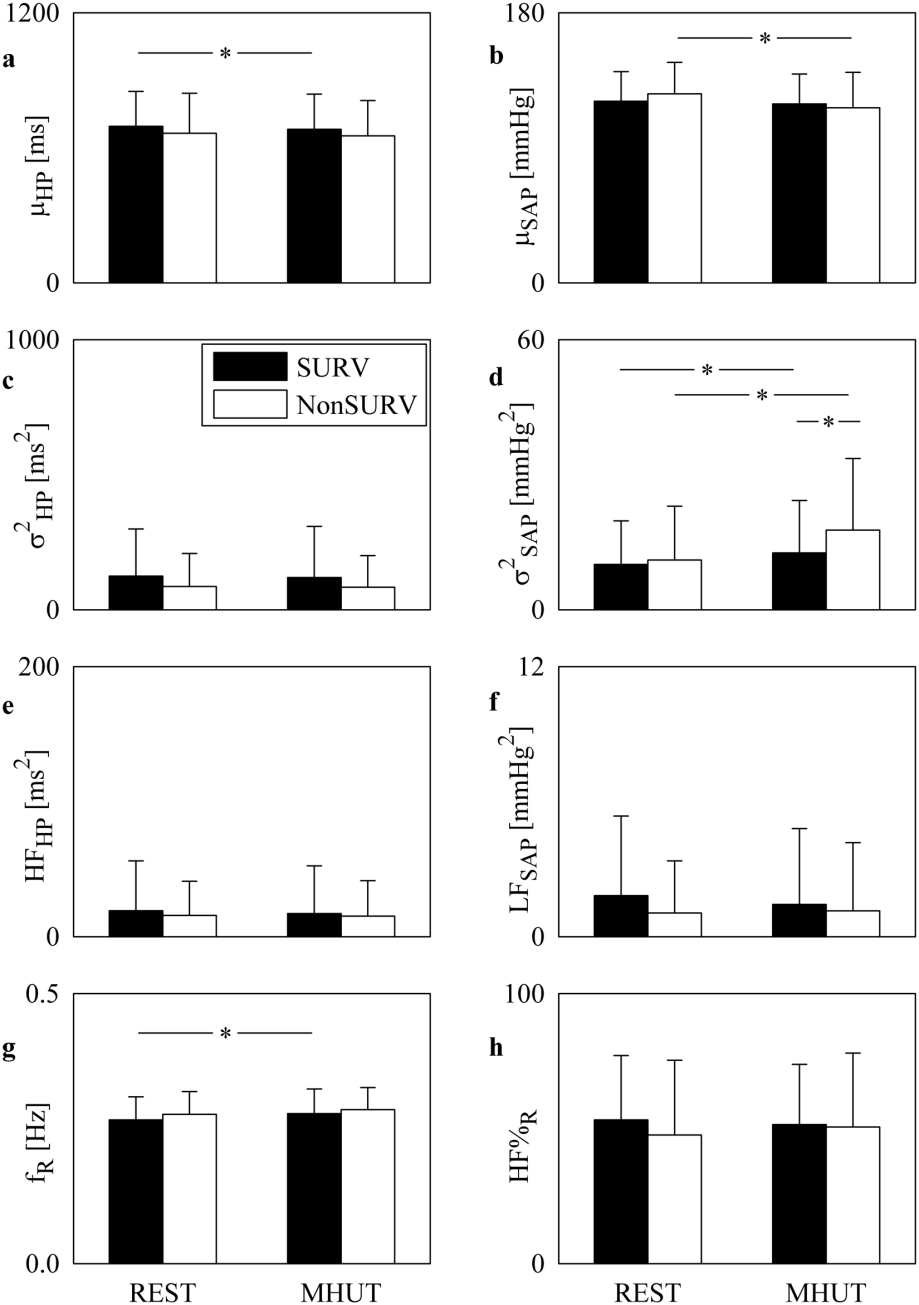
The bar graphs show μ_HP_ (**a**), μ_SAP_ (**b**), σ^2^_HP_ (**c**), σ^2^_SAP_ (**d**), HF_HP_ (**e**), LF_SAP_ (**f**), f_R_ (**g**) and HF%_R_ (**h**) as a function of the experimental condition (i.e. REST and MHUT) in SURVs (dark bar) and NonSURVs (white bar). Data are reported as mean plus standard deviation. The symbol *indicates *p* < 0.05.

Figure 1 shows the bar graphs relevant to univariate time and frequency domain indexes such as μ_HP_ (Fig. 1a), μ_SAP_ (Fig. 1b), σ^2^_HP_ (Fig. 1c), σ^2^_SAP_ (Fig. 1d), HF_HP_ (Fig. 1e), LF_SAP_ (Fig. 1f), f_R_ (Fig. 1g) and HF%_R_ (Fig. 1h). Over HP series significant differences were limited: indeed, only μ_HP_ decreased significantly during MHUT in SURVs (Fig. 1a). Over SAP series, μ_SAP_ decreased significantly during MHUT in NonSURVs (Fig. 1b) and σ^2^_SAP_ increased significantly during MHUT in both SURVs and NonSURVs but the rise was more important in NonSURVs (Fig. 1d). Similarly to the frequency domain indexes computed over HP series (i.e. HF_HP_, Fig. 1e) also the frequency domain marker computed over SAP series (i.e. LF_SAP_, Fig. 1f) did not differentiate groups and conditions. Over R series f_R_ increased significantly during MHUT in SURVs (Fig. 1g), while HF%_R_ did not vary with group and condition (Fig. 1h).

**Figure 2:**
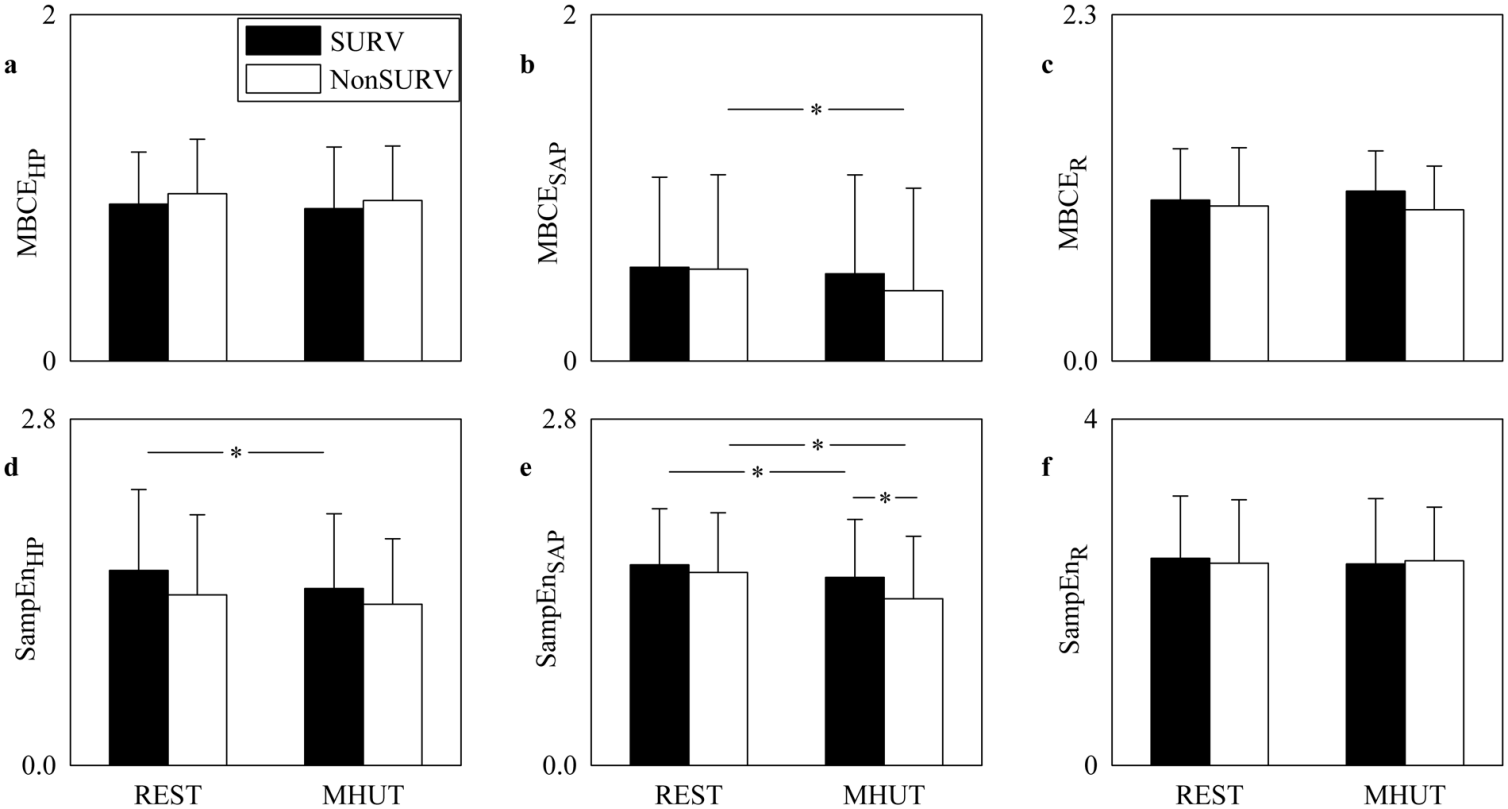
The bar graphs show MBCE and SampEn computed on HP series in (**a**) and (**d**) respectively, on SAP series in (**b**) and (**e**) respectively, and on R series in (**c**) and (**f**) respectively, as a function of the experimental condition (i.e. REST and MHUT) in SURVs (dark bar) and NonSURVs (white bar). Data are reported as mean plus standard deviation. The symbol *indicates *p* < 0.05.

Figure 2 shows the bar graphs relevant to univariate complexity analysis reporting MBCE and SampEn as computed over HP series (Fig. 2a,d), over SAP series (Fig. 2b,e), and R series (Fig. 2c,f). The bar graphs have the same structure as in Fig. 1. The linear index of complexity (i.e. MBCE) was unable to differentiate groups and conditions regardless of the series with the notable exception of MBCE_SAP_ that decreased during MHUT in NonSURVs (Fig. 2b). SampEn_HP_ decreased significantly during MHUT in SURVs (Fig. 2d). The effect of MHUT was not visible in NonSURVs (Fig. 2d), mainly because in NonSURVs SampEn_HP_ was small at REST (Fig. 2d). SampEn_SAP_ decreased significantly during MHUT in both SURVs and NonSURVs. During orthostatic challenge SampEn_SAP_ was significantly smaller in NonSURVs than in SURVs (Fig. 2e). SampEn_R_ did not distinguish either experimental condition or group (Fig. 2f).

**Figure 3:**
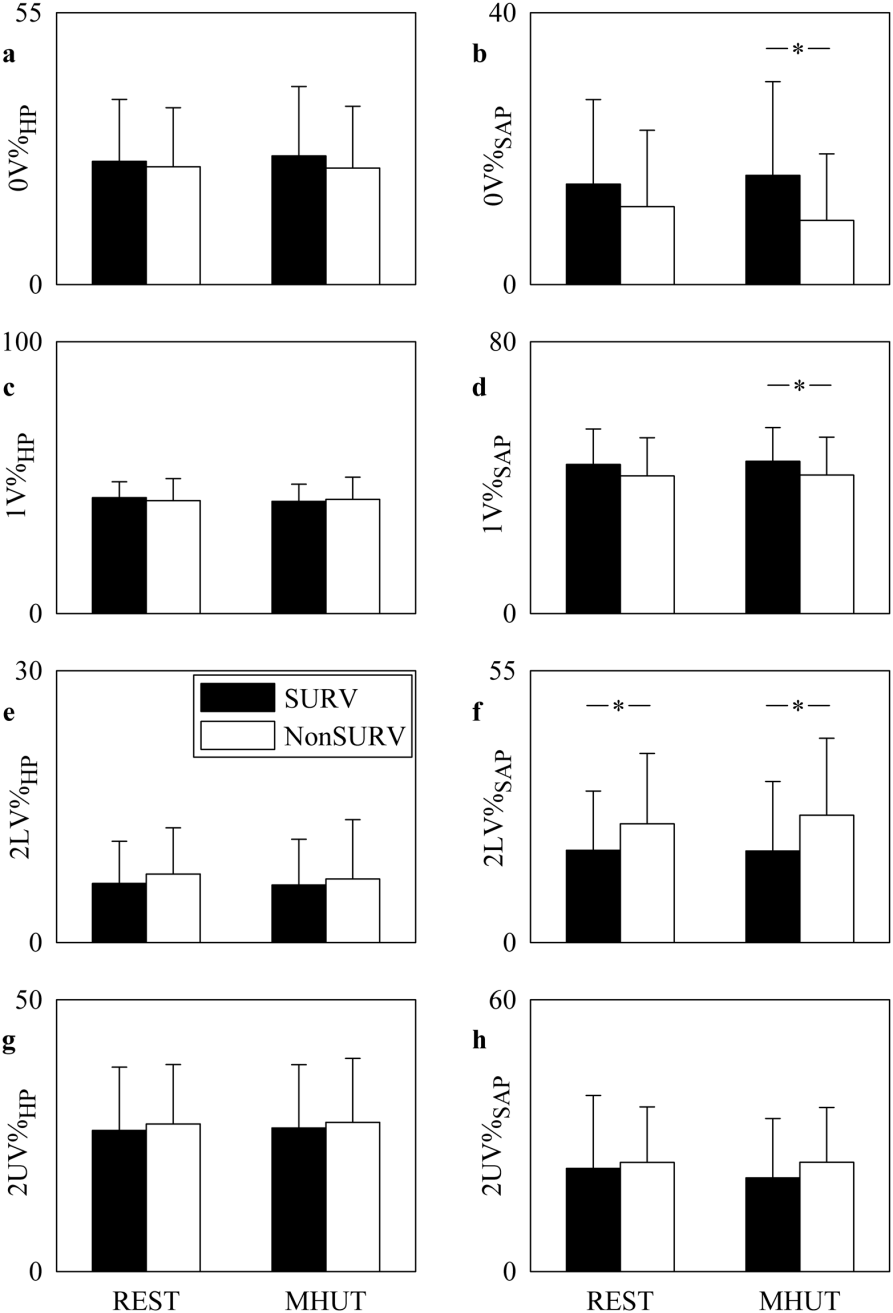
The bar graphs show 0 V% (**a**,**b**), 1 V% (**c**,**d**), 2LV% (**e**,**f**) and 2UV% (**g**,**h**) computed over HP (**a**,**c**,**e**,**g**) and SAP (**b**,**d**,**f**,**h**) series as a function of the experimental condition (i.e. REST and MHUT) in SURVs (dark bar) and NonSURVs (white bar). Data are reported as mean plus standard deviation. The symbol *indicates *p* < 0.05.

Figure 3 shows the bar graphs relevant to univariate symbolic analysis markers as computed over HP (Fig. 3a,c,e,g) and SAP (Fig. 3b,d,f,h) series. 0 V% (Fig. 3a,b), 1V% (Fig. 3c,d), 2LV% (Fig. 3e,f) and 2UV% (Fig. 3g,h) are reported. The bar graphs have the same structure as in Fig. 1. None of the univariate symbolic analysis indexes computed over HP series distinguished groups and conditions (Fig. 3a,c,e,g). 0 V%_SAP_, 1 V%_SAP_, 2LV%_SAP_ and 2UV%_SAP_ were not affected by MHUT regardless of the group (Fig. 3b,d,f,h). During MHUT 0 V%_SAP_ and 1 V%_SAP_ decreased in NonSURVs compared to SURVs (Fig. 3b,d), while both at REST and during MHUT 2LV%_SAP_ increased (Fig. 3f). 2UV%_SAP_ did not vary between groups and conditions (Fig. 3h).

**Figure 4:**
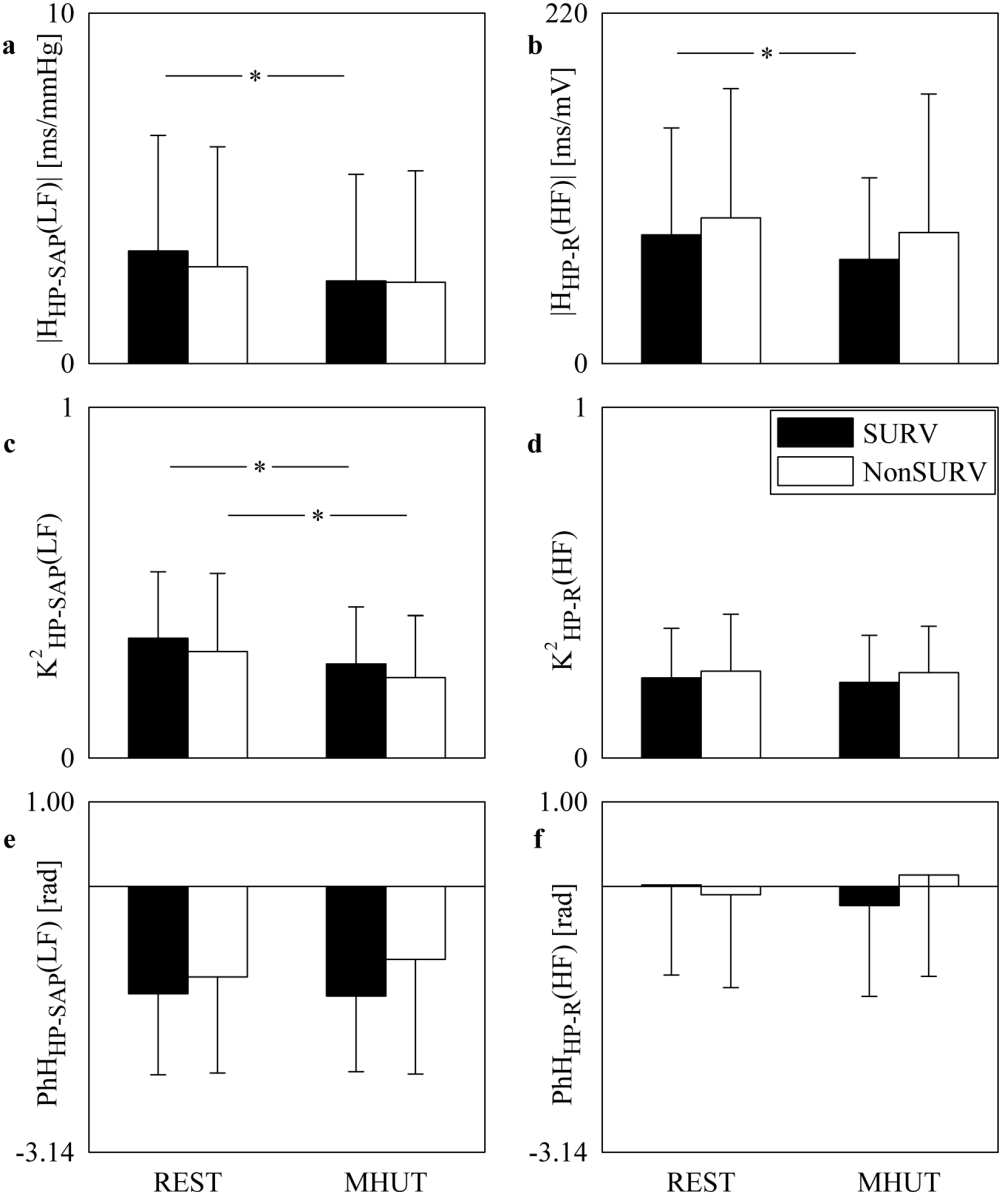
The bar graphs show |H_HP-SAP_(LF)| (**a**), |H_HP-R_(HF)| (**b**), K^2^_HP-SAP_(LF) (**c**), K^2^_HP-R_(HF) (**d**), PhH_HP-SAP_(LF) (**e**), and PhH_HP-R_(HF) (**f**) as a function of the experimental condition (i.e. REST and MHUT) in SURVs (dark bar) and NonSURVs (white bar). Data are reported as mean plus standard deviation. The symbol *indicates *p* < 0.05.

Figure 4 shows the bar graphs relevant to cross-spectral analysis between HP and SAP series (Fig. 4a,c,e) and between HP and R (Fig. 4b,d,f). |H_HP-SAP_(LF)| (Fig. 4a), K^2^_HP-SAP_(LF) (Fig. 4c), PhH_HP-SAP_(LF) (Fig. 4e),|H_HP-R_(HF)| (Fig. 4b), K^2^_HP-R_(HF) (Fig. 4d), and PhH_HP-R_(HF) (Fig. 4f) are shown. The bar graphs have the same structure as in Fig. 1.|H_HP-SAP_(LF)| and |H_HP-R_(HF)| decreased during MHUT solely in SURVs (Fig. 4a,b) and K^2^_HP-SAP_(LF) diminished in both SURVs and NonSURVs (Fig. 4c). K^2^_HP-R_(HF), PhH_HP-SAP_(LF) and PhH_HP-R_(HF) were stable with group and experimental condition (Fig. 4d,e,f).

**Figure 5:**
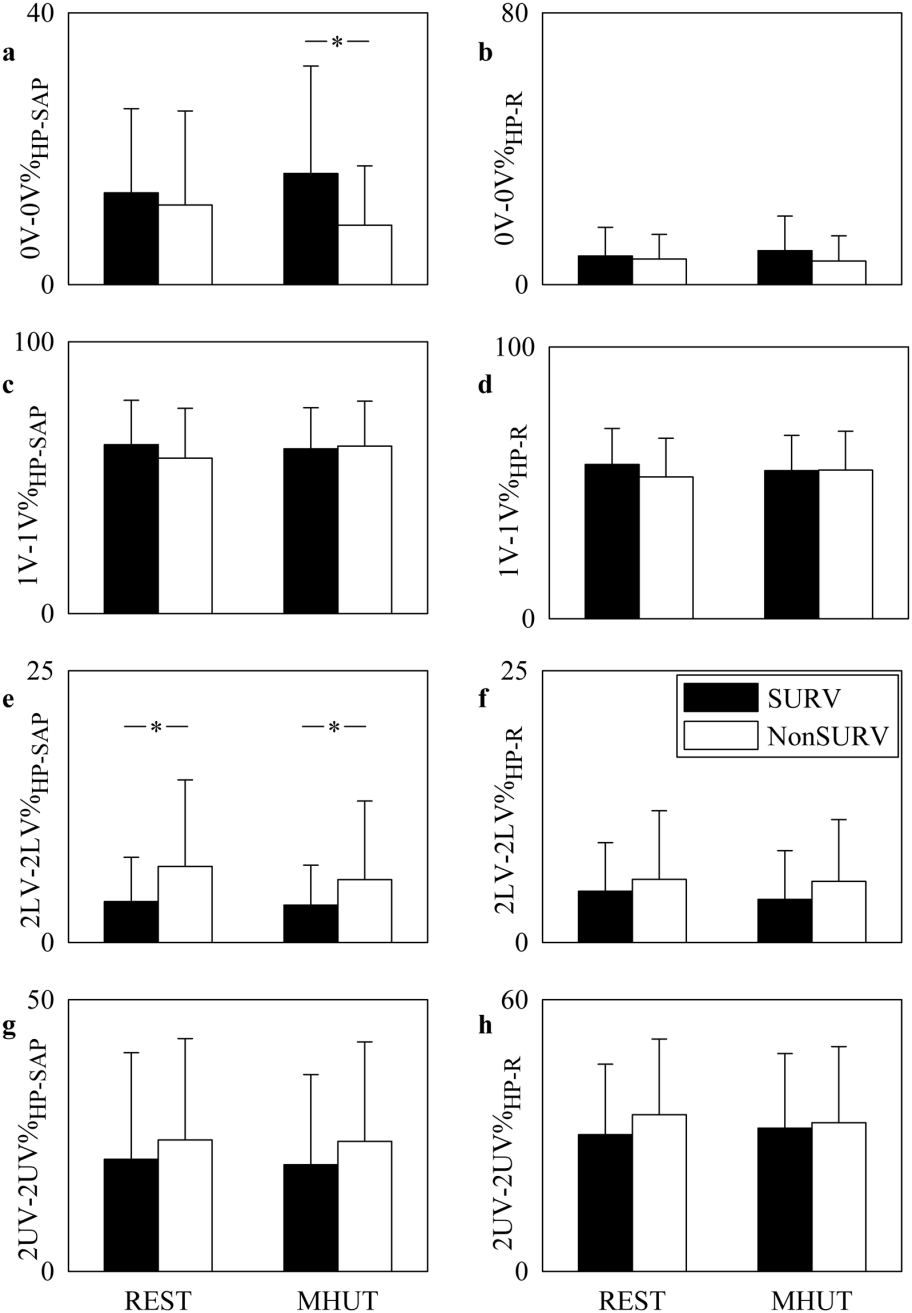
The bar graphs show 0V-0V% (**a**,**b**), 1V-1V% (**c**,**d**), 2LV-2LV% (**e**,**f**) and 2UV-2UV%(**g**,**h**) computed to typify the dynamical interactions between HP and SAP series (a,c,e,g) and between HP and R (**b**,**d**,**f**,**h**) as a function of the experimental condition (i.e. REST and MHUT) in SURVs (dark bar) and NonSURVs (white bar). Data are reported as mean plus standard deviation. The symbol *indicates *p* < 0.05.

Figure 5 shows the bar graphs relevant to the bivariate joint symbolic analysis between HP and SAP series (Fig. 5a,c,e,g) and between HP and R series (Fig. 5b,d,f,h). 0V-0V% (Fig. 5a,b), 1V-1V% (Fig. 5c,d), 2LV-2LV% (Fig. 5e,f) and 2UV-2UV% (Fig. 5g,h) are shown. The bar graphs have the same structure as in Fig. 1. None of the bivariate joint symbolic analysis markers describing the dynamical interactions between HP and R series varied with group and condition (Fig. 5b,d,f,h). Conversely, 0V-0V%_HP-SAP_ differentiated NonSURVs from SURVs during MHUT (Fig. 5a) and 2LV-2LV%_HP-SAP_ distinguished NonSURVs from SURVs both at REST and during MHUT (Fig. 5e). 1V-1V%_HP-SAP_ and 2UV-2UV%_HP-SAP_ remained stable within groups and conditions (Fig. 5c,g).

**Figure 6:**
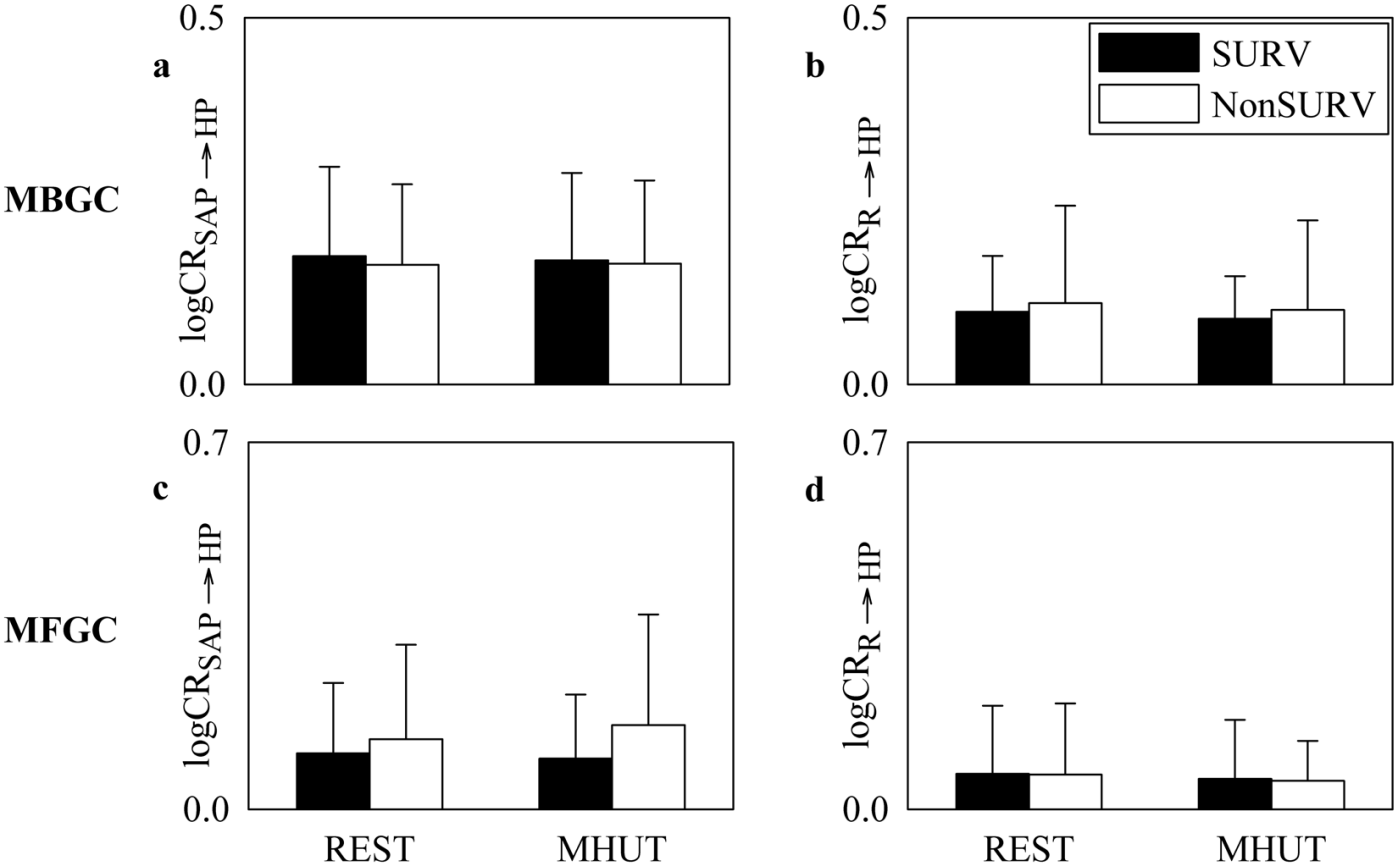
The bar graphs show $\mathrm{log}\,{{\rm{CR}}}_{{\rm{SAP}}\to {\rm{HP}}}$ (**a**,**c**) and $\mathrm{log}\,{{\rm{CR}}}_{{\rm{R}}\to {\rm{HP}}}$ (**b**,**d**) computed via MBGC (**a**,**b**) and MFGC (**c**,**d**) methods as a function of the experimental condition (i.e. REST and MHUT) in SURVs (dark bar) and NonSURVs (white bar). Data are reported as mean plus standard deviation.

Figure 6 shows the results of MBGC (Fig. 6a,b) and MFGC (Fig. 6c,d) analyses carried out along the cardiac baroreflex from SAP to HP using logCR_SAP_ _→_ _HP_ (Fig. 6a,c) and along the cardiorespiratory pathway from R to HP using logCR_R_ _→_ _HP_ (Fig. 6b,d). The bar graphs have the same structure as in Fig. 1. The analyses were always made in Ω = {HP,SAP,R}, thus disambiguating eventual influences of the third variable different from the source and the destination. Both markers did not vary with groups and conditions and this conclusion held regardless of the method (i.e. MBGC or MFGC approach).

Results of univariate Cox regression analysis carried out over variables separating SURVs from NonSURVs are reported in Table 2. The variables undergoing univariate Cox regression analysis were the presence of septic shock, SOFA score, SAPS II, 2LV%_SAP_ and 2LV-2LV%_HP-SAP_ at REST, σ^2^_SAP_, SampEn_SAP_, 0 V%_SAP_, 1 V%_SAP_, 2LV%_SAP_, 0V-0V%_HP-SAP_ and 2LV-2LV%_HP-SAP_ during MHUT. All variables were found to be significantly associated with mortality (Table 2). When these parameters entered in a multivariate Cox regression model, only SAPS II and σ^2^_SAP_ during MHUT carried complementary information with type I error probability, hazard risk and 95% confidence interval, respectively, equal to 3.9 × 10^−4^, 1.04, 1.018–1.062 and 2.6 × 10^−3^, 1.029, 1.010–1.049.

**Table 2:** Results of univariate Cox regression analysis.

Variable	Type I error probability	Hazard risk	95% confidence interval
Septic shock	0.033	2.037	1.059–3.916
SOFA score	0.011	1.089	1.020–1.162
SAPS II	0.001	1.034	1.013–1.055
2LV%_SAP_ at REST	0.039	1.024	1.001–1.046
2LV-2LV%_HP-SAP_ at REST	0.001	1.069	1.027–1.113
σ^2^_SAP_ during MHUT	0.031	1.022	1.004–1.041
SampEn_SAP_ during MHUT	0.021	0.471	0.249–0.893
0 V%_SAP_ during MHUT	0.019	0.96	0.928–0.993
1 V%_SAP_ during MHUT	0.029	0.972	0.947–0.997
2LV%_SAP_ during MHUT	0.01	1.025	1.006–1.045
0V-0V%_HP-SAP_ during MHUT	0.036	0.965	0.933–0.998
2LV-2LV%_HP-SAP_ during MHUT	0.011	1.066	1.015–1.120

SOFA = sepsi-related organ failure assessment; SAPS = simplified acute physiology score; REST = at rest in supine position; MHUT = modified head-up tilt; HP = heart period; SAP = systolic arterial pressure; σ^2^_SAP_ = variance of SAP; SampEn_SAP_ = sample entropy of SAP; 0 V%_SAP_, 1 V%_SAP_ and 2LV%_SAP_ = percentage of 0 V, 1 V, and 2LV patterns respectively as detected by univariate symbolic analysis of SAP; 0V-0V%_HP-SAP_ and 2LV-2LV%_HP-SAP_ = percentage of 0V-0V and 2LV-2LV patterns respectively as detected by bivariate symbolic analysis of HP and SAP.

## Discussion

The main findings of the study can be summarized as follows: i) autonomic markers assessed during the first day after admission in ICU were associated with mortality; ii) even though the association was visible at REST, the exploitation of an orthostatic challenge, such as MHUT, amplified the possibility offered by autonomic markers; iii) SAP variability indexes were more likely to be associated with mortality, while HP variability indexes were useless; iv) nonlinear indexes computed via model-free approaches (i.e. symbolic analysis and SampEn) were more powerful than linear ones in stratifying the risk of patients in ICU; v) while markers derived from the HP-SAP variability interactions appeared to be helpful, those assessing HP-R variability relation were useless; vi) causality markers along cardiac baroreflex and cardiopulmonary pathway were not linked to the mortality and this conclusion held regardless of the method (i.e. MBGC or MFGC); vii) when all indexes showing a significant association with mortality were tested together in a multivariate Cox regression model, only SAPS II and SAP variance during MHUT carried complementary information.

### Study hypotheses and rationale underlying autonomic challenge in ICU

We hypothesized that cardiovascular and cardiorespiratory control indexes closely linked to the autonomic nervous system state could be valuable candidates to be included in a model predicting the risk of mortality in patients admitted to ICU and that an orthostatic maneuver operated at patient’s bedside in the ICU could be fruitfully exploited to unveil the association of autonomic markers with mortality. If SURV and NonSURV groups could be separated according to one of the proposed autonomic control markers, that parameter should be a valuable candidate in a multivariate clinical model predicting the risk of mortality for people admitted to ICU and its incremental value to clinical scores traditionally utilized in ICU, such as the SOFA score and SAPS II, should be tested. The separation between SURVs and NonSURVs cannot be trivially assumed given that individuals admitted to ICU might exhibit an impaired autonomic control in relation to their pathological state and might have undergone a pharmacological treatment, such as the administration of catecholamines, and/or an intervention, such as sedation, interfering with the ability of autonomic function to govern physiological variables. Given the presumed low level of residual ability of the autonomic nervous system to regulate physiological variables in critical patients, we supposed that the application of an autonomic challenge such as the MHUT^38^ could be helpful to amplify the difference between those subjects who preserved autonomic control and those who did not. However, if the separation could be obtained just at REST, the candidate variable should be considered more powerful than the one leading to a separation solely during MHUT given that no stressor is needed, thus simplifying the test procedure.

### Distinguishing SURVs from NonSURVs during their first day in ICU

We found that autonomic nervous system markers, although depressed in both SURVs and NonSURVs, can be fruitfully exploited to distinguish the two groups and MHUT can favor this separation. The list of the parameters allowing this distinction comprises σ^2^_SAP_, SampEn_SAP_, 0 V%_SAP_, 1 V%_SAP_, 0V-0V%_HP-SAP_ solely during MHUT, 2LV%_SAP_ and 2LV-2LV%_HP-SAP_ both at REST and during MHUT. Since more variables distinguished SURVs from NonSURVs during MHUT than at REST, we conclude that an orthostatic maneuver carried out at the patient’s bedside is helpful toward the stratification of the mortality risk in people admitted to ICU. We also observed that SAP variability is more helpful than the HP one. Indeed, none of the variability markers involving HP series could distinguish NonSURVs from SURVs. This result stresses the relevance of analyzing SAP series in addition to the HP one^51^. The variance of SAP during MHUT was significantly larger in NonSURVs than in SURVs, thus indicating a greater difficulty of NonSURVs in keeping under control SAP fluctuations and a reduced capacity of NonSURVs in coping with external and/or internal disturbances. Also symbolic analysis of SAP variability evidenced that SAP values are less stable in NonSURVs than in SURVs: as a matter of fact, the likelihood of more stable SAP patterns decreased and the rate of more variable SAP features increased in NonSURVs. Complexity indexes describing the HP-SAP variability interactions via joint symbolic analysis showed a remarkable power in distinguishing SURVs from NonSURVs. Indeed, during MHUT the HP-SAP coupling at slow time scales, as monitored via 0V-0V%_HP-SAP_, was significantly smaller in NonSURVs than in SURVs, while the opposite situation was observed at faster time scales as indicated by the trend of 2LV-2LV%_HP-SAP_. This finding once again emphasized the pathological response of NonSURVs to the orthostatic stimulus that usually leads in healthy population to an increase of 0V-0V%_HP-SAP_ and a decrease of 2LV-2LV%_HP-SAP_ as an indication of the activation of the cardiac baroreflex^52^.

Remarkably, nonlinear model-free markers appear to be more powerful than linear model-based ones in distinguishing SURVs from NonSURVs. For example, complexity markers computed via SampEn were more powerful than those calculated via MBCE approach and univariate symbolic indexes were more effective than spectral ones. A model-free approach based on joint symbolic analysis was able to separate the two groups, while linear model-based methods assessing the gain and phase of the transfer function and Granger causality indexes could not. Moreover, the list of parameters helpful in distinguishing SURVs from NonSURVs stresses that the more elaborated the method, the weaker its ability in separating SURVs from NonSURVs. Indeed, markers of cardiac baroreflex, assessing the HP-SAP gain, HP-SAP phase and strength of global association between HP and SAP, and indexes of causality along cardiac baroreflex, estimating the strength of the linear association in the temporal direction from SAP to HP, could not distinguish SURVs from NonSURVs and, at the best, could detect the effect of MHUT. The same consideration holds for the analysis of HP-R variability interactions. This observation is in line with a recent contribution^14^ suggesting that in clinical applications and in pathological groups the robustness of the index could be more important than its methodological design. At this regard joint symbolic analysis might provide a good tradeoff between the complexity of the approach allowing the description of dynamical interactions among physiological variables and the robustness of the method.

### Association of cardiovascular control indexes with mortality and their complementary value to traditional risk scores

All cardiovascular control markers able to differentiate SURVs from NonSURVs were found significantly associated with mortality via univariate Cox regression analysis, thus suggesting that they can be potentially helpful in stratifying the mortality risk of critical patients admitted to ICU. However, only σ^2^_SAP_ during MHUT carried complementary information to traditional risk indicators that were able to distinguish the two groups as well (i.e. the presence of septic shock, SOFA score and SAPS II). This result stressed the relevance of monitoring short-term SAP variability and of applying an autonomic challenge in ICU. The hypovolemic condition evoked by MHUT^38^ is likely to have an impact much stronger in NonSURVs who could not appropriately deal with it, as suggested by the larger amplitude of SAP oscillations that were not efficiently buffered due to a more impaired cardiovascular control. We recommend the application of this autonomic challenge to gain additional information and the inclusion of σ^2^_SAP_ measured during the challenge in mortality risk scores.

### Effects of MHUT in patients during their first day in ICU

MHUT induces an increase of SAP fluctuations in the LF band and a decrease of cardiac baroreflex sensitivity in healthy subjects with age similar to those of the present study, being these findings compatible with a sympathetic activation directed to the vessel and with a cardiac baroreflex unloading associated with the challenge^38^. In healthy subjects the central hypovolemia evoked by an orthostatic challenge led to an increased strength of the HP-SAP variability relation along cardiac baroreflex^37,52,53^, likely related to the augmented involvement of this reflex in preserving adequate arterial pressure levels^54,55^, a decreased strength of the cardiorespiratory coupling^45,53^ and a diminished complexity of cardiac control^32,56^, likely related to the vagal withdrawal associated with the challenge^54,57–59^. Conversely, the complexity of vascular control in healthy individuals was not affected by the orthostatic challenge^56,60^ likely because arterial pressure regulatory mechanisms are not under vagal control. Remarkably, in SURVs a subset of these modifications in response to MHUT could be observed, thus suggesting that autonomic control preserved a certain ability to govern the changes of physiological variables in this subgroup of critically ill individuals. For example, cardiac baroreflex sensitivity and complexity of HP series decreased significantly in SURVs during MHUT. Conversely, the decrease of the amount of linear association between HP and SAP in a frequency band typical of the baroreflex functioning (i.e. LF band) during MHUT, observed in both SURVs and NonSURVs, suggests an impairment of the cardiovascular control in both groups and a greater isolation of physiological systems typical of an organism that lost its ability to provide an integrated response to a stressor. An additional sign of the autonomic control impairment in both SURVs and NonSURVs is the inability of observing the expected modifications of the strength of causal relation along cardiac baroreflex and cardiopulmonary pathway during MHUT^37,45,53^. MHUT highlighted some peculiar differences between SURVs and NonSURVs. For example, the larger increase of the magnitude of the SAP variability during MHUT in NonSURVs should be interpreted as a greater inability of the regulatory systems in this group in limiting arterial pressure instabilities compared to SURVs more than a sign of the sympathetic activation evoked by the stimulus. In addition, the decrease of complexity of the vascular control observed only in NonSURVs indicated an excessive simplification of the mechanisms of arterial pressure regulation, thus stressed again the limited regulatory resources of this group.

## Conclusions

The stratification of mortality risk at the admission in ICU is commonly carried out by several scores that do not account for autonomic control markers. The rationale underlying this choice is that autonomic control mechanisms might be impaired and/or depressed in the category of individuals admitted to ICU as a consequence of their pathological condition and/or pharmacological intervention to such a level that the information carried by autonomic control indexes might be negligible or meaningless. Conversely, this study suggests that markers of autonomic regulation computed over SAP variability are associated with mortality in patients admitted to ICU and this association is even stronger whether an orthostatic challenge is applied at the bedside to stimulate an autonomic reaction. More specifically, we recommend the application of an autonomic challenge at the bedside in ICU and the inclusion of the amplitude of SAP variability into more traditional scores of mortality risk given its complementary value.

### Data availability

Data are fully available through the corresponding author.

## Electronic supplementary material

Supplementary Information
