# Supplementary material for: Association between autonomic control indexes and mortality in subjects admitted to intensive care unit
Source: Sci Rep. 2018 Feb 22;8:3486. doi: 10.1038/s41598-018-21888-8 (PMC5823868; doi:10.1038/s41598-018-21888-8)
Supplement: Supplementary file 1 — Supplementary Information [file 41598_2018_21888_MOESM1_ESM.doc]

Supplementary material of the MS “Association between autonomic control indexes and mortality in subjects admitted to intensive care unit” by Alberto Porta, Riccardo Colombo, Andrea Marchi, Vlasta Bari, Beatrice De Maria, Giovanni Ranuzzi, Stefano Guzzetti, Tommaso Fossali, and Ferdinando Raimondi

**S.1. Model-based frequency domain analysis**

Spectral analysis was performed via a model-based parametric approach exploiting the autoregressive (AR) model^1^. Briefly, the AR model describes the zero-mean series *y*={*y_n_*, *n*=1, …, *N*}, where *n* is the progressive sample counter and *N* is the series length, as

$y_{n}=\sum_{k=1}^{d} \text{a}_{k}\cdot y_{n-k}+w_{n}$ (1),

where *a_k_* with *k*=1, …, *d* are real coefficients and *w* is a realization of zero-mean Gaussian white noise with variance *λ*^2^. The series *y* is the sum of two parts: i) a fully predictable portion modeled as a linear combination of *d* past samples weighted by real coefficients; ii) a fully unpredictable portion described by *w*. The equivalent representation of the AR process in the *z*-domain allows one to write the *z*-transformation of the AR process as the product of the *z*-transformation of *w* by the function

$H(z)=\frac{1}{1-\sum_{k=1}^{d} a_{k}\cdot z^{-k}}=\frac{z^{d}}{\prod_{k=1}^{d} (z-p_{k})}$, (2)

referred to as transfer function of the AR process, featuring *d* singularities *p_k_*=*ρ*e^j^*^φ^* with *k*=1, …, *d* called poles. Given that the coefficients of the AR process are real, if a complex pole (i.e. *p_k_*=*ρ*e^j^*^φ^* with *φ*≠0 and *φ*≠π) is present also *p_k_*=*ρ*e^-j^*^φ^* is found. The Levinson-Durbin recursive algorithm^2^ was utilized to estimate directly from *y*, the coefficients *a_k_* with *k*=1, …, *d* and *λ*^2^. According to the maximum entropy spectral estimation approach, the power spectral density *S*(*f*) can be computed from *H*(*z*) and *λ*^2^ as

$S(f)=\left.T\cdot H\text{(}z\text{)}\cdot H\text{(}z^{-1}\text{)}\cdot\lambda^{2}\right|_{z=\text{e}^{\text{j2π}fT}}$, (3)

where *T* is the sampling period and j is the imaginary unit in the complex plane. *S*(*f*) can be factorized in a sum of terms^1^, referred to as spectral components that, transformed back in the time domain, correspond to basic AR processes associated to a real pole or a pair of complex and conjugate poles of *H*(*z*). The sum of the power of all components is equal to the variance of the AR process. A spectral component was attributed to a given frequency band if the phase *φ* of the associated real pole or pair of complex and conjugate poles, when converted into a frequency *f* using the transformation *f*=*φ*∙(2π*T*)^-1^, dropped in that frequency band. The total power in a given frequency band was computed as the sum of the powers of all spectral components attributed to that band.

**S.2. Model-based linear complexity analysis**

We quantified complexity through the computation of conditional entropy (CE). CE is a measure of the amount of information carried by the most recent sample *y_n_* of the pattern ***y****_d_*,*_n_*=(*y_n_*, *y_n_*_-1_,…, *y_n_*-*_d_*_+1_) of length *d* that cannot be derived from its *d*-1 past samples [i.e. $\mathbf{y}_{d,n}^{-}=(y_{n-1},\cdot\cdot\cdot,y_{n-d+1})$]. CE is a measure of randomness given that it is null in presence of fully predictable signals, while it is maximum when *y* is fully unpredictable (i.e. white noise). If *y* is an AR process of order *d* as described by (1), CE is given as^3^

$\text{CE}(\left.y_{n}\right|\mathbf{y}_{d,n}^{-})=0.5\cdot\log(2\text{πe}\lambda^{2})$, (4)

where log is the natural logarithm, e is the Euler’s number and *λ*^2^ can be estimated as the variance of the one-step-ahead prediction error

, (5)

where $\hat{a}_{k}$ with *k*=1, …, *d* are the AR coefficients estimated via e.g. the least squares procedure^2^.

**S.3. Model-free complexity analysis**

The majority of model-free CE estimators are grounded on the observation that^4^

$\text{CE}(\left.y_{n}\right|\mathbf{y}_{d,n}^{-})=\text{ShE}(\mathbf{y}_{d,n})-\text{ShE}(\mathbf{y}_{d,n}^{-})$, (6)

where $\text{ShE}(\mathbf{y}_{d,n})=-\sum\text{p(}\mathbf{y}_{d,n}\text{)}\cdot\log[\text{p(}\mathbf{y}_{d,n})]$ and $\text{ShE}(\mathbf{y}_{d,n}^{-})=-\sum\text{p(}\mathbf{y}_{d,n}^{-}\text{)}\cdot\log[\text{p(}\mathbf{y}_{d,n}^{-})]$ are the amount of information carried by ***y****_d_*,*_n_* and of $\mathbf{y}_{d,n}^{-}$ respectively with p(***y****_d_*,*_n_*) and $p(\mathbf{y}_{d,n}^{-})$ denoting the probability of ***y****_d_*,*_n_* and $\mathbf{y}_{d,n}^{-}$ respectively. Among the possible methods for model-free CE estimation^3^, we selected the sample entropy (SampEn)^5^. In SampEn computation the $\text{ShE}(\mathbf{y}_{d,n})$ and $\text{ShE}(\mathbf{y}_{d,n}^{-})$ in (6) were computed as –log<p(***y****_d_*,*_n_*)> and –log<$p(\mathbf{y}_{d,n}^{-})$>,where <·> takes the average over the time index *n*, and p(***y****_d_*,*_n_*) and $p(\mathbf{y}_{d,n}^{-})$ were estimated as the fraction of patterns at distance closer than *r* from the reference patterns ***y****_d_*,*_n_* and $\mathbf{y}_{d,n}^{-}$ respectively. The parameter *r* set the tolerance for the assessment of pattern similarity under a given metric (here the Euclidean norm). Patterns at zero distance from the reference pattern (i.e. self-matches) were excluded from the neighbors of the reference pattern and, thus they were not counted in the estimation of p(***y****_d_*,*_n_*) and $p(\mathbf{y}_{d,n}^{-})$.

**S.4. Univariate symbolic analysis**

Univariate symbolic analysis was carried out according to the uniform quantization and redundancy reduction approach set in Porta et al^6^. Briefly, the series of length *N* was transformed into a sequence of *ξ* symbols via the uniform quantization procedure consisting of coarse graining the min-max range of the series with *ξ* equal-size bins and substituting the values inside each bin with an integer ranging from 0 to *ξ*-1 coding the bin. The symbolic series was converted into a series of patterns formed by *d* consecutive delayed symbolic values with superposition between adjacent patterns. Given *ξ*, *d* and *N*, the number of possible patterns is *ξ^d^*, while the number of patterns really detected in *N*-*d*+1. The typical choice of ξ=6 and d=3 allowed us one to fulfill the relation *ξ^d^*<*N*-*L*+1 suggesting that the rate of occurrence of a given pattern was reliably estimated^6^. According to the complexity of the described feature the symbolic patterns were classified into four classes: (i) no variation (0V); (ii) one variation (1V); (iii) two like variations (2LV); and (iv) two unlike variations (2UV). The 0V pattern had the lowest level of complexity because all symbols were equal, whereas the 2UV pattern featured the highest level of complexity because all symbols were different compared with the previous one and variations between two consecutive original values were of opposite sign. 1V and 2LV patterns exhibited an intermediate level of complexity given that in the 1V pattern only two consecutive symbols were equal and in the 2LV pattern all symbols were different, but variations between two consecutive original values were of the same sign. The 2LV pattern was more variable than the 1V one owing to the higher variability of symbols. Because any symbolic pattern of length *d* was univocally associated with one of the four classes, the sum of the number of 0V, 1V, 2LV and 2UV patterns was *N*−*d*+1 and the percentage of 0V, 1V, 2LV and 2UV patterns (i.e. 0V%, 1V%, 2LV% and 2UV%) could be computed.

**S.5. Model-based cross-spectral analysis**

Cross-spectral analysis of *y*_1_={*y*_1_,*_n_*, *n*=1, …, *N*} and *y*_2_={*y*_2_,*_n_*, *n*=1, …, *N*} was performed via a model-based parametric approach exploiting the bivariate AR model^1,7^. Briefly, the bivariate AR model jointly describes *y*_1_ and *y*_2_ as

$y_{1,n}=\sum_{k=1}^{d} a_{11,k}\cdot y_{1,n-k}+\sum_{k=\tau_{12}}^{d} a_{12,k}\cdot y_{2,n-k}+w_{1,n}$ (7)

$y_{2,n}=\sum_{k=\tau_{21}}^{d} a_{21,k}\cdot y_{1,n-k}+\sum_{k=1}^{d} a_{22,k}\cdot y_{2,n-k}+w_{2,n}$, (8)

where *a*_11_,*_k_* and *a*_22_,*_k_* with *k*=1, …, *d* are the real coefficients of the auto-regressions of *y*_1_ and *y*_2_ on its own past respectively, *a*_12_,*_k_* with *k*=*τ*_12_, …, *d* and *a*_21_,*_k_* with *k*=*τ*_21_, …, *d* are the real coefficients of the cross-regressions of *y*_1_ on past values of *y*_2_ and *vice versa*, and *w*_1_ and *w*_2_ are two uncorrelated zero-mean white noises with variance *λ*^2^_1_ and *λ*^2^_2_ respectively. *τ*_12_ and *τ*_21_ represent the delay of interactions from *y*_2_ to *y*_1_ and from *y*_1_ to *y*_2_ respectively. The equivalent representation of the bivariate AR process ***y***=|*y*_1_ *y*_2_|′ in the *z*-domain allows one to provide the *z*-transformation of ***y*** as the product the *z*-transformation of the white noise ***w***=|*w*_1_ *w*_2_|′ by the transfer function matrix
 ***H***(*z*)=(**I**-**A**(z))^-1^, where **I** is the identity 2x2 matrix and

$\mathbf{A}\text{(}z)=\left|\begin{matrix} \sum_{k=1}^{d} a_{11,k}\cdot z^{-k} & \sum_{k=\tau_{12}}^{d} a_{12,k}\cdot z^{-k} \\ \sum_{k=\tau_{21}}^{d} a_{21,k}\cdot z^{-k} & \sum_{k=1}^{d} a_{22,k}\cdot z^{-k} \end{matrix}\right|$ (9)

is the 2x2 matrix of polynomials in *z* and the symbol ′ denotes the transpose operator. The coefficients of ***A***(*z*) are estimated directly from ***y*** via the traditional least squares approach solved using Cholesky decomposition method^1,8^. The number of useful past samples *d* is usually fixed *a priori* according to methodological considerations about spectral resolution or optimized using some figure of merit (i.e. the Akaike criterion^9^) and the delays *τ*_12_ and *τ*_21_ were usually set^6^ to 1. According to the maximum entropy spectral estimation approach, power spectral density matrix ***S***(*f*) can be computed from ***H***(*z*) and the covariance matrix ***Λ*** of ***w*** as

. (10)

***S***(*f*) features the power spectral densities of *y*_1_ and *y*_2_, *S*_11_(*f*) and *S*_22_(*f*), on the main diagonal and the cross-spectral densities from *y*_2_ to *y*_1_ and from *y*_1_ to *y*_2_, *S*_12_(*f*) and *S*_21_(*f*), out of the main diagonal. While *S*_11_(*f*) and *S*_22_(*f*) are real functions of *f*, cross-spectral densities are complex functions of *f* with *S*_12_(*f*)=*S^*^*_21_(*f*), where the symbol * denotes the complex conjugation operator. The transfer function from *y*_2_ to *y*_1_ is *H*_12_(*f*)=*S*_12_(*f*)/*S*_22_(*f*). The transfer function modulus from *y*_2_ to *y*_1_ is |*H*_12_(*f*)|=|*S*_12_(*f*)|/*S*_22_(*f*), where |·| takes the modulus of *S*_12_(*f*). The phase of *H*_12_(*f*) ranges from –π to + π and it is known at multiples of 2π. This means that, if the phase of *H*_12_(*f*) is ∠*H*_12_(*f*), also ∠*H*_12_(*f*)+2π*k* with *k*=0, ±1, ±2, … is admissible leading to the ambiguous conversion of the phase values into delays or advancements^10^ and making phase analysis useless to infer causality without suing *a priori* information about the latency of interactions^10^. Squared coherence function *K*^2^_12_(*f*)=|*S*_12_(*f*)|2/[*S*_11_(*f*)·*S*_22_(*f*)] is a measure of the strength of linear association between *y*_1_ and *y*_2_ as a function of *f*. *K*^2^_12_(*f*) ranges from 0 to 1, where 0 indicates null and maximum linear association between *y*_1_ and *y*_2_. Given that *K*^2^_12_(*f*) is a symmetric function [i.e. *K*^2^_12_(*f*)=*K*^2^_21_(*f*)], *K*^2^_12_(*f*) is useless to assess the strength of the linear association between *y*_1_ and *y*_2_ in an assigned direction of interactions^11^.

**S.6. Joint symbolic analysis**

At difference with the model-based cross-spectral analysis described in the Sect.S.5 “Model-based cross-spectral analysis” joint symbolic analysis can describe nonlinear interactions among series. We exploited the approach set in Porta et al^12^. Briefly, given two, contemporaneously recorded, series the univariate symbolic approach described in the Sect.S.4 “Univariate symbolic analysis” was first applied to both series, thus obtaining two sequences of *N*−*d*+1 symbolic patterns of length *d*. The interactions between the two series were studied by building joint schemes formed by a symbolic pattern of the first series and the pattern of second series *τ* time steps ahead that of the first one. Coordinated behaviors were investigated by searching for joint schemes formed by symbolic patterns belonging to the same family labeled as 0V-0V, 1V-1V, 2LV-2LV and 2UV-2UV. The percentage of 0V-0V, 1V-1V, 2LV-2LV and 2UV-2UV patterns (i.e. 0V-0V%, 1V-1V%,
2LV-2LV% and 2UV-2UV%) was computed by dividing their number by the total number of coordinated patterns and, then, by multiplying the result by 100.

**S.7. Linear model-based Granger causality (MBGC) analysis**

MBGC^13^ approach provides the chance of assessing the amount of association between *y_i_* and *y_j_* with *i*≠*j* in an assigned causal direction (e.g. from *y_j_* to *y_i_*), while disambiguating the possible influences of confounding factors described by signals different from *y_i_* and *y_j_*. According to the Granger causality principle, given a set of *M* zero-mean signals *Ω*={*y*_1_, …, *y_i_*, …, *y_j_*, …, *y_M_*} with *y_i_*={*y_i_*,*_n_*, *n*=1, …, *N*} and *y_j_*={*y_j_*,*_n_*, *n*=1, …, *N*} with *i*,*j*=1, …, *M*, usually termed as full universe of knowledge in the Granger causality terminology, *y_j_* Granger-causes *y_i_*, usually indicated as *y_j_*→*y_i_*, if *y_i_* can be better predicted in the full *Ω* than in the restricted *Ω* obtained from *Ω* after excluding the presumed cause *y_j_* (i.e. *Ω*\*y_j_*). Therefore, the practical implementation of the Granger causality principle requires the comparison between the predictions of *y_i_* in the full and restricted *Ω*s. Under the hypothesis that *y_i_* can be modeled as an AR process with *M*-1 exogenous inputs *y_l_* (ARX) with *l*=1, …, *M* and *l*≠*i*

$y_{i,n}=\sum_{k=1}^{d} \left.a_{ii,k}\right|_{\Omega}\cdot y_{i,n-k}+\sum_{l=1,l\neq i}^{M} \sum_{k=\tau_{il}}^{d} \left.a_{il,k}\right|_{\Omega}\cdot y_{l,n-k}+\left.w_{i,n}\right|{}_{\Omega}$ (11)

provides the description of the dynamics of *y_i_* in *Ω* and

$y_{i,n}=\sum_{k=1}^{d} \left.a_{ii,k}\right|_{\Omega\backslash y_{j}}\cdot y_{i,n-k}+\sum_{l=1,l\neq i,j}^{M} \sum_{k=\tau_{il}}^{d} \left.a_{il,k}\right|_{\Omega\backslash y_{j}}\cdot y_{l,n-k}+\left.w_{i,n}\right|{}_{\Omega\backslash y_{j}}$ (12)

the analogous description of *y_i_* in *Ω*\*y_j_*. The ARX coefficients *a_ii_*,*_n_* and *a_il_*,*_n_* with *l*≠*i* in *Ω* and *Ω*\*y_j_*, can be estimated via traditional least squares procedure using the Cholesly decomposition method, the number *d* of past samples utilized in the auto- and cross-regressions can be selected via an optimization criterion (e.g. the Akaike figure of merit for multivariate ARX process^9^) applied to the ARX model in *Ω* and, then, extended to *Ω*\*y_j_*^14^ and the delay *τ_ij_* of interactions *a priori* set in agreement with methodological considerations and measurement conventions, is maintained both in *Ω* and *Ω*\*y_j_*^14^. The one-step-ahead prediction of *y_i_*,*_n_* in *Ω* and *Ω*\*y_j_*, indicated as and respectively, can be deterministically obtained by regressing the signals present in *Ω* and *Ω*\*y_j_* according to the estimated coefficients $\hat{a}_{ii,n}$ and $\hat{a}_{il,n}$ with *l*≠*i* in *Ω* as

(13)

and in *Ω*\*y_j_* as

. (14)

Defined the prediction errors in *Ω* and in *Ω*\*y_j_*, $\left.e_{i,n}\right|_{\Omega}$ and $\left.e_{i,n}\right|_{\Omega\backslash y_{j}}$, as the difference between *y_i_*,*_n_* and in *Ω* and between *y_i_*,*_n_* and in *Ω*\*y_j_*, the variances of the prediction errors, $\left.e_{i}^{2}\right|_{\Omega}$ and $\left.e_{i}^{2}\right|_{\Omega\backslash y_{j}}$, quantify the inability of the ARX model in predicting *y_i_* in the full and restricted *Ω*s respectively. The larger the $\left.e_{i}^{2}\right|_{\Omega}$ and $\left.e_{i}^{2}\right|_{\Omega\backslash y_{j}}$, the most unpredictable the *y_i_* in *Ω* and *Ω*\*y_j_* respectively. Since the zero-mean signals in *Ω* are normalized to have unit variance, $\left.e_{i}^{2}\right|_{\Omega}$ and $\left.e_{i}^{2}\right|_{\Omega\backslash y_{j}}$ are bounded below 1. The comparison between $\left.e_{i}^{2}\right|_{\Omega}$ and $\left.e_{i}^{2}\right|_{\Omega\backslash y_{j}}$ is carried out via the log-causality ratio (logCR)

$\log\text{CR}_{y_{j}\to y_{i}}=\log\left(\frac{\left.e_{i}^{2}\right|_{\Omega\backslash y_{j}}}{\left.e_{i}^{2}\right|_{\Omega}}\right)$, (15)

assessing the unpredictability decrement due to the inclusion of the presumed cause *y_j_* in *Ω*\*y_j_*. Among the possible causality indexes^15^ the logCR has the advantage of providing an intrinsic normalization for the difficulty in predicting *y_i_*.

**S.8. Model-free Granger causality (MFGC) analysis**

The principle underlying Granger causality makes the approach independent of the method utilized to assess the relation of *y_i_* on its own past values and past values of all the remaining signals in the full and restricted *Ω*s. Therefore, a MFGC approach such as that based on k-nearest neighbor strategy can be fruitfully exploited^16^. Defined the multivariate embedding vector ***z****_i_*,*_n_*=(***y****^i^*_1_,*_n_*, …, ***y****^i^_i_*,*_n_*, …, ***y****^i^_j_*,*_n_*, …, ***y****^i^_M_*,*_n_*) with *i*=1, …, *M* formed by the univariate embedding vectors ***y****^i^_j_*,*_n_*=($y_{j,n-\tau_{ij}}$, …, ) with *j*=1, …, *M* The MFGC approach hypothesizes that there is a function *f*(·) in *Ω*, $\left.f(\cdot)\right|_{\Omega}$, linking ***z****_i_*,*_n_* to *y_i_*,*_n_* in the form $y_{i,n}=\left.f(\mathbf{z}_{i,n})\right|_{\Omega}+\varepsilon_{i,n}$, where *ε_i_*,*_n_* describes the unpredictable part of *y_i_*,*_n_*. The k-nearest neighbor method provides an estimate of the *f*(·), $\hat{f}(\cdot)$, that allows one to obtain as follows^17^: defined as *y_i_*,*_n_* the image of the reference vector ***z****_i_*,*_n_*, is simply obtained by a suitable average^17^ of the images of the k nearest neighbors of ***z****_i_*,*_n_*. Nonuniform embedding strategies were exploited for the construction of ***z****_i_*,*_n_*. They were based on a construction of a set of candidate samples formed by components of the signals present in *Ω* with time indexes less recent than *n*. Starting from an initially void multivariate embedding vector, candidates samples were tested once at time for their inclusion in the multivariate embedding vector. The candidate sample leading to the most significant increment of prediction ability was included in the multivariate embedding vector^18,19,20^. During the construction of the multivariate embedding space values of the series more recent than those already selected were excluded. The incremental procedure for the construction of the embedding space allowed the optimization of *τ_ij_* and *d_ij_*. Prediction ability was assessed^17^ as the correlation between *y_i_* and . The optimal multivariate embedding space was the one leading to the maximum improvement of predictability and its dimension was the optimal multivariate embedding dimension^18^. The exclusion of the components of the presumed cause from the optimal multivariate embedding vector allowed us to compute . From and the prediction errors $\left.e_{i,n}\right|_{\Omega}$ and $\left.e_{i,n}\right|_{\Omega\backslash y_{j}}$ could be derived and, finally, from their variances $\left.e_{i}^{2}\right|_{\Omega}$ and $\left.e_{i}^{2}\right|_{\Omega\backslash y_{j}}$ we calculated $\log\text{CR}_{y_{j}\to y_{i}}$.

**References**

1. Baselli, G., Porta, A., Rimoldi, O., Pagani, M. & Cerutti S. Spectral decomposition in multichannel recordings based on multi-variate parametric identification. *IEEE Trans. Biomed. Eng.* **44**, 1092-1101 (1997).

2. Kay, S.M. & Marple, S.L. Spectrum analysis: a modern perspective. *Proc. IEEE* **69**, 1380-1418, (1981).

3. Porta, A., De Maria, B., Bari, V., Marchi, A. & Faes, L. Are nonlinear model-free conditional entropy approaches for the assessment of cardiac control complexity superior to the linear model-based one?. *IEEE Trans. Biomed. Eng.* **64**, 1287-1296 (2017).

4. Richman, J.S. & Moorman, J.R. Physiological time-series analysis using approximate entropy and sample entropy. *Amer. J. Physiol.* **278**, H2039-H2049 (2000).

5. McEliece, R.J. *The Theory of Information and Coding* (Cambridge University Press, Cambridge, UK, 2002).

6. Porta, A., Guzzetti, S., Montano, N., Furlan, R., Malliani, A. & Cerutti, S. Entropy, entropy rate and pattern classification as tools to typify complexity in short heart period variability series. *IEEE Trans. Biomed. Eng.* **48**, 1282-1291 (2001).

7. Porta, A., Baselli, G., Rimoldi, O., Malliani, A. & Pagani, M. Assessing baroreflex gain from spontaneous variability in conscious dogs: role of causality and respiration. *Am. J. Physiol.* **279**, H2558-H2567 (2000).

8. Söderström, T. & Stoica, P. *System Identification* (Prentice Hall International, Englewood Cliffs, UK, 1989).

9. Akaike, H. A new look at the statistical novel identification. *IEEE Trans. Autom. Control* **19**, 716-723 (1974).

10. Porta, A., Catai, A.M., Takahashi, A.C.M., Magagnin, V., Bassani, T., Tobaldini, E., van de Borne, P. & Montano, N. Causal relationships between heart period and systolic arterial pressure during graded head-up tilt. *Am. J. Physiol.* **300**, R378-R386 (2011).

11. Porta, A., Furlan, R., Rimoldi, O., Pagani, M., Malliani, A. & van de Borne, P. Quantifying the strength of linear causal coupling in closed loop interacting cardiovascular variability series. *Biol. Cybern.* **86**, 241-251 (2002).

12. Porta, A., Marchi, A., Bari, V., Heusser, K., Tank, J., Jordan, J., Barbic, F. & Furlan, R. Conditional symbolic analysis detects non linear influences of respiration on cardiovascular control in humans, *Phil. Trans. R. Soc. A* **373**, 20140096 (2015).

13. Granger, C.W.J. Testing for causality. A personal viewpoint. *J. Econ. Dyn. Control* **2**, 329-352 (1980).

14. Porta, A. & Faes, L. Wiener-Granger causality in network physiology with applications to cardiovascular control and neuroscience. *Proc. IEEE* **104**, 282-309 (2016a).

15. Porta, A., Bari, V., Marchi, A., De Maria, B., Takahashi, A.C.M., Guzzetti, S., Colombo, R., Catai, A.M. & Raimondi, F. Effect of variations of the complexity of the target variable on the assessment of Wiener-Granger causality in cardiovascular control studies. *Physiol. Meas.* **37**, 276-290 (2016b).

16. Farmer, J.D. & Sidorowich, J.J. Predicting chaotic time series. *Phys. Rev. Lett.* **59**, 845-848 (1987).

17. Porta, A., Guzzetti, S., Furlan, R., Gnecchi-Ruscone, T., Montano, N. & Malliani A. Complexity and nonlinearity in short-term heart period variability: comparison of methods based on local nonlinear prediction. *IEEE Trans. Biomed. Eng.* **54**, 94-106 (2007).

18. Porta, A., Faes, L., Bari, V., Marchi, A., Bassani, T., Nollo, G., Perseguini, N.M., Milan, J., Minatel, V., Borghi-Silva, A., Takahashi, A.C.M. & Catai, A.M. Effect of age on complexity and causality of the cardiovascular control: comparison between model-based and model-free approaches. *PLoS ONE* **9**, e89463 (2014).

19. Vlachos, I. & Kugiumtzis, D. Nonuniform state-space reconstruction and coupling direction. *Phys. Rev. E* **82**, 016207 (2010).

20. Faes, L., Nollo, N. & Porta, A. Information based detection of nonlinear Granger causality in multivariate processes via a nonuniform embedding technique. *Phys. Rev. E* **83**, 051112 (2011).
